# Integrative Transcriptomic and Single-Cell Protein Characterization of Colorectal Carcinoma Delineates Distinct Tumor Immune Microenvironments Associated with Overall Survival

**DOI:** 10.21203/rs.3.rs-4751101/v1

**Published:** 2024-07-25

**Authors:** Erika Hissong, Bhavneet Bhinder, Junbum Kim, Kentaro Ohara, Hiranmayi Ravichandran, Majd Al Assaad, Sarah Elsoukkary, Michael Shusterman, Uqba Khan, Kenneth Wha Eng, Rohan Bareja, Jyothi Manohar, Michael Sigouros, Andre F. Rendeiro, Jose Jessurun, Allyson J. Ocean, Andrea Sboner, Olivier Elemento, Juan Miguel Mosquera, Manish A. Shah

**Affiliations:** Department of Pathology and Laboratory Medicine, Weill Cornell Medicine, 525 E 68th St, New York, NY 10065; Englander Institute for Precision Medicine, Weill Cornell Medicine and New York Presbyterian, 413 E 69th St, New York, NY 10021, USA; Englander Institute for Precision Medicine, Weill Cornell Medicine and New York Presbyterian, 413 E 69th St, New York, NY 10021, USA; Englander Institute for Precision Medicine, Weill Cornell Medicine and New York Presbyterian, 413 E 69th St, New York, NY 10021, USA; Department of Pathology and Laboratory Medicine, Weill Cornell Medicine, 525 E 68th St, New York, NY 10065; Englander Institute for Precision Medicine, Weill Cornell Medicine and New York Presbyterian, 413 E 69th St, New York, NY 10021, USA; Englander Institute for Precision Medicine, Weill Cornell Medicine and New York Presbyterian, 413 E 69th St, New York, NY 10021, USA; Department of Pathology and Laboratory Medicine, Weill Cornell Medicine, 525 E 68th St, New York, NY 10065; Englander Institute for Precision Medicine, Weill Cornell Medicine and New York Presbyterian, 413 E 69th St, New York, NY 10021, USA; Department of Pathology and Laboratory Medicine, Weill Cornell Medicine, 525 E 68th St, New York, NY 10065, USA; Department of Medicine, Hematology and Medical Oncology, Weill Cornell Medicine, 525 E 68th St New York, NY, USA; Department of Medicine, Hematology and Medical Oncology, Weill Cornell Medicine, 525 E 68th St New York, NY, USA; Englander Institute for Precision Medicine, Weill Cornell Medicine and New York Presbyterian, 413 E 69th St, New York, NY 10021, USA; Englander Institute for Precision Medicine, Weill Cornell Medicine and New York Presbyterian, 413 E 69th St, New York, NY 10021, USA; Englander Institute for Precision Medicine, Weill Cornell Medicine and New York Presbyterian, 413 E 69th St, New York, NY 10021, USA; Englander Institute for Precision Medicine, Weill Cornell Medicine and New York Presbyterian, 413 E 69th St, New York, NY 10021, USA; Englander Institute for Precision Medicine, Weill Cornell Medicine and New York Presbyterian, 413 E 69th St, New York, NY 10021, USA; CeMM Research Center for Molecular Medicine of the Austrian Academy of Sciences, Lazarettgasse 14, AKH BT 25.3, 1090 Wien, Austria; Department of Pathology and Laboratory Medicine, Weill Cornell Medicine, 525 E 68th St, New York, NY 10065, USA; Englander Institute for Precision Medicine, Weill Cornell Medicine and New York Presbyterian, 413 E 69th St, New York, NY 10021, USA; Department of Medicine, Hematology and Medical Oncology, Weill Cornell Medicine, 525 E 68th St New York, NY, USA; Department of Pathology and Laboratory Medicine, Weill Cornell Medicine, 525 E 68th St, New York, NY 10065; Englander Institute for Precision Medicine, Weill Cornell Medicine and New York Presbyterian, 413 E 69th St, New York, NY 10021, USA; Institute for Computational Biomedicine, Weill Cornell Medicine, 525 E 68th St. New York, NY, USA; Englander Institute for Precision Medicine, Weill Cornell Medicine and New York Presbyterian, 413 E 69th St, New York, NY 10021, USA; Department of Physiology and Biophysics, Weill Cornell Medicine, 525 E 68th St. New York, NY, USA; Institute for Computational Biomedicine, Weill Cornell Medicine, 525 E 68th St. New York, NY, USA; Department of Pathology and Laboratory Medicine, Weill Cornell Medicine, 525 E 68th St, New York, NY 10065; Englander Institute for Precision Medicine, Weill Cornell Medicine and New York Presbyterian, 413 E 69th St, New York, NY 10021, USA; Englander Institute for Precision Medicine, Weill Cornell Medicine and New York Presbyterian, 413 E 69th St, New York, NY 10021, USA; Department of Medicine, Hematology and Medical Oncology, Weill Cornell Medicine, 525 E 68th St New York, NY, USA

**Keywords:** colorectal cancer, mass cytometry, tumor microenvironment

## Abstract

Colorectal carcinoma (CRC) is a heterogeneous group of tumors with varying therapeutic response and prognosis, and evidence suggests the tumor immune microenvironment (TIME) plays a pivotal role. Using advanced molecular and spatial biology technologies, we aimed to evaluate the TIME in patients with CRC to determine whether specific alterations in the immune composition correlated with prognosis.

We identified primary and metastatic tumor samples from 31 consented patients, which were profiled with whole-exome sequencing and bulk RNA-seq. Immune cell deconvolution followed by gene set enrichment analysis and unsupervised clustering was performed. A subset of tumors underwent *in situ* analysis of the TIME spatial composition at single-cell resolution through Imaging Mass Mass Cytometry.

Gene set enrichment analysis revealed two distinct groups of advanced CRC, one with an immune activated phenotype and the other with a suppressed immune microenvironment. The activated TIME phenotype contained increased Th1 cells, activated dendritic cells, tertiary lymphoid structures, and higher counts of CD8+ T cells whereas the inactive or suppressed TIME contained increased macrophages and a higher M2/M1 ratio. Our findings were further supported by RNA-seq data analysis from the TCGA CRC database, in which unsupervised clustering also identified two separate groups. The immunosuppressed CRC TIME had a lower overall survival probability (HR 1.66, p=0.007).

This study supports the pertinent role of the CRC immune microenvironment in tumor progression and patient prognosis. We characterized the immune cell composition to better understand the complexity and vital role that immune activity states of the TIME play in determining patient outcome.

## Introduction

Despite advances in screening and early therapeutic interventions, colorectal carcinoma remains a significant cause of morbidity and is one of the leading causes of cancer-related deaths in both men and in women [1]. Up to one-fourth of patients present with metastatic disease at the time of diagnosis and nearly half of patients who have had curable intent resection will still develop recurrence [2–4]. Importantly, colorectal cancer (CRC) is a heterogeneous disease with a diverse molecular background that has significant implications for therapeutic approaches and overall survival. Indeed, unique tumor subsets have specific therapeutic vulnerabilities, such as RAS mutant,mismatch repair deficiency,, or HER2 overexpression.. Advances in the field of molecular pathology have greatly facilitated our understanding of the genomic and epigenomic landscape of CRC, with extensive work done by consortia such as The Cancer Genome Atlas (TCGA) and the international consensus molecular subtype classification consortium, which has enabled the classification of various subtypes of CRC according to their distinct molecular profiles and clinicopathologic features [5,6]. This genomic heterogeneity may partially explain the observation that the aggressiveness of metastatic CRC varies widely among patients, with some harboring localized, resectable metastases that can be managed with curative intent whereas others progress rapidly.

Beyond tumor molecular profiling alone, substantial evidence has highlighted the importance of the tumor immune microenvironment (TIME) in CRC, with some authors suggesting that immune features might serve as a better prognostic indicator than traditional histopathologic methods [7,8]. An example of this, the immunoscore, developed by Galon *et al.* and based on the quantification of CD3 and CD8-positive T cells using digital imaging analysis, was found to outperform the Tumor-Node-Metastases (TNM) classification for disease-free survival and overall survival and could help predict response to therapy [9–11]. Other studies have found that mature T cells, dendritic cells, and memory T cells were associated with improved prognosis whereas regulatory T cells and M2 macrophages were associated with inferior prognosis [12,13]. Since these initial pivotal studies, our ability to assess the TIME has significantly improved, which has led to a better understanding of its complexity. Recent advances in whole slide scanning, multiplexed single-cell *in situ* protein profiling and machine learning algorithms have enabled more objective quantification. Additionally, single-cell technologies including RNA sequencing have led to further insights into the TIME [14,15].

The purpose of this study was to evaluate the TIME in patients with CRC to determine whether specific alterations in the immune composition correlated with histopathologic features, molecular alterations, and overall prognosis. We found that CRC TIME could be characterized as either immune activated (higher proportion of activated dendritic cells, tertiary lymphoid structures, higher CD8 + T cells) or inactivated (inactivated dendritic cells, lower CD8 + T cells, increased M2 macrophages), and that the activated immune microenvironment is associated with improved survival.

## Materials and Methods

### Case selection and histopathology review

We included all patients with colorectal carcinoma who had been consented for molecular testing through the Englander Institute for Precision Medicine at Weill Cornell Medicine [16]. Each sample, including primary and metastatic site, was reviewed by a certified pathologist to confirm the histologic diagnosis. Only fresh frozen samples with sufficient RNA content and quality were included in the study cohort. Clinicopathologic data as well as therapeutic interventions and follow-up information was obtained through review of available surgical pathology material and the electronic medical record. This study was approved by the institutional review board (IRB) at Weill Cornell Medicine (IRB protocol #1305013903).

### Whole-exome and bulk RNA sequencing

As described in detail in prior publications [16,17], both tumor and germline DNA were extracted using the Promega Maxwell 16 MDx DNA Purification Kits (Promega, Madison, WI). Whole-exome sequencing (WES) was performed using the Illumina HiSeq2500 (2×100bp) (Illumina, San Diego, CA). Reads were aligned to the GRCh37/hg19 reference and processed according to the Whole Exome Sequencing Test for Cancer - ExaCT1 pipeline v0.9 [16–18]. Total RNA was extracted for RNAseq using Promega Maxwell 16 MDx instrument. RNA integrity was verified using the Agilent Bioanalyzer 2100 (Agilent Technologies) and prepared for whole transcriptome analysis (RNA-Seq) in accordance with the standard Illumina mRNA sample preparation protocol (Illumina, San Diego, CA). Paired-end RNA-Seq at read lengths of 50 base pairs was performed with the HiSeq 2000 (Illumina). We performed sequence alignment using STAR_2.4 with human genome sequence build hg19, downloaded via the UCSC genome browser (http://hgdownload.soe.ucsc.edu/goldenPath/hg19/bigZips/).

### Immune cell deconvolution and TCGA cohort validation

We utilized xCell, a gene signature-based algorithm, for the deconvolution of bulk RNA-sequencing data to interrogate the tumor immune landscape [19]. We evaluated transcriptome expression data for 64 immune and stromal cell types and the results were associated with WES results. Consensus clustering was performing using ConsensusClusterPlus R package. To validate our findings, the TCGA colon cancer cohort [6] (n = 477) was also interrogated, and the results were correlated with Consensus Molecular Subtypes of CRC (https://portal.gdc.cancer.gov).

### Immunohistochemistry (IHC)

Cases were re-reviewed by a certified pathologist with subspecialty training in gastrointestinal pathology. Histologic features including tumor architecture, tumor-stroma interface, immune infiltration, and stromal response were recorded for each case. On a subset of tumors IHC was performed on sections of formalin-fixed paraffin-embedded (FFPE) tumor tissue using a Bond III automated immunostainer and the Bond Polymer Refine detection system (Leica Microsystems, IL, USA). The following antibodies and conditions (dilution, antigen retrieval solution and pH, antigen retrieval time) were used: CD86 (CellSignaling Technology E2G8P Rabbit mAb, 1:100, Sodium Citrate buffer, pH6, 30 min) and CD80 (MyBioSource MBS4380739 Mouse mAb, 1:100, Sodium Citrate buffer, pH6, 30 min).

### Imaging Mass Cytometry

Per the manufacturer’s protocol, antibodies were conjugated in BSA and Azide-free format using the MaxPar X8 multimetal labeling kit (Standard BioTool). The antibodies were tested on control tissues (*e.g*., lymph node, tonsil) to validate the staining pattern as verified by study pathologists. Fifteen tumors (2 primary and 13 metastases) from 13 patients underwent *in situ* immune evaluation by imaging mass cytometry (IMC). The tissue samples were stained according to the manufacturer protocol following traditional immunohistochemical methods. Briefly, after 2 hours of warming the slides, these were deparaffinized in Citrisolv twice for 15 mins each followed by ethanol rehydration. Antigen retrieval was performed in a water bath for 30 minutes at pH 9.0. The slides were then washed and blocked using ThermoFischer SuperBlock solution for an hour. The slides were incubated overnight in antibody cocktail and washed the next day in TBS buffer and water. Lastly, DNA intercalator staining was performed by incubating the slides in 1:400 Ir solution for 30 mins at RT. The slides were washed and air-dried, ready to be scanned. Two regions of interest (ROI) were chosen per tumor, including tumor/normal interface when present.

### Imaging Mass Cytometry Data Analysis

The IMC^™^ data was processed using the imc (v.0.1.4) package, available at [https://github.com/ElementoLab/imc] as we previously reported [20,21]. The 29-channel image was reduced into a probability map of nuclei, cytoplasm, and background. Cells were segmented by applying DeepCell (v.0.2.1) to the probability map. To identify the expression profiles in the multiplexed images, each channel of every cell was mean-aggregated. The Scanpy library was utilized for downstream preprocessing. A log transformation and z-score normalization truncated at 3 standard deviations was performed to bring all channels to the same scale. This was followed by batch correction using Harmony (v.0.0.9) [10.5281/zenodo.7351719] to mitigate sample-specific batch effects. To identify cell phenotypes, leiden clustering was applied with a resolution of 1.0[22]. Each resulting cluster was manually labeled using the matrixplot provided. For statistical analysis, the cellular density per ROI was calculated and a two-sided Mann-Whitney U-test was conducted, followed by a Benjamini-Hochberg multiple hypothesis correction to account for the repeated comparisons across cell types.

## Results

### Clinico-pathologic characteristics are representative of conventional colorectal carcinoma

Forty-one (41) colorectal carcinoma specimens from 31 unique patients (M:F ratio = 21:10, average age 55 years) were included in the study cohort (Table 1). Most (58%) were left-sided colorectal carcinomas. At the time of tissue acquisition, all but 3 patients had Stage IV disease, and most (77%) patients had previously received first-line chemotherapy prior to tissue collection. Nine samples (9) were from the primary tumor and 32 samples were taken from metastatic sites. The molecular characteristics of this cohort were fairly representative of conventional colorectal carcinoma, with frequent alterations in KRAS (74%), TP53 (71%), and APC (51%); The majority of tumors (95%) were microsatellite stable. Consensus molecular subtypes 2 and 4 were most common (27% and 24%, respectively). The follow up interval from date of diagnosis to last contact was 56 months (range 4–168 mo). Twenty-two (70%) patients were alive, and the mean survival for the cohort was 40 mo (range 21–70 months).

### Colorectal Cancer is characterized by two clusters with distinct immune environments

**Figure 1:**
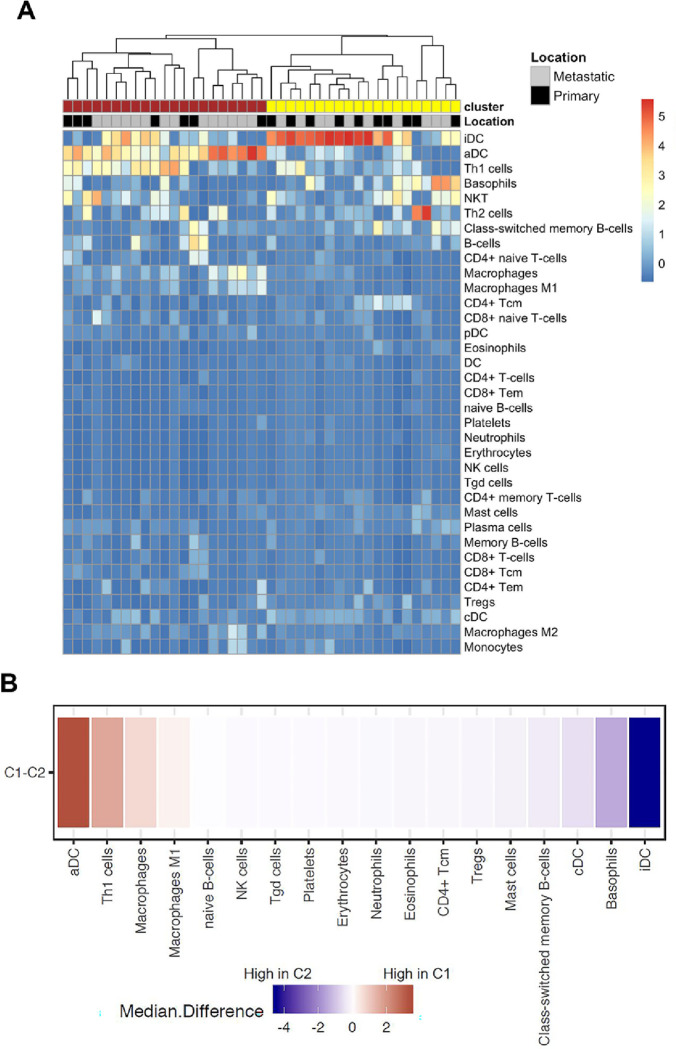
**a.** Deconvolved immune cell types from RNA-Seq data classify colorectal cancer samples into two broad clusters with distinct immune environments (C1-red, C2-yellow). **b.** Cluster C1 contains a higher number of activated dendritic cells and Th1 cells (activated immune microenvironment), whereas cluster C2 contains increased inactive dendritic cells and basophils (inactivated immune microenvironment).

Gene set enrichment analysis followed by unsupervised clustering revealed two distinct clusters, which varied in their immune composition (Fig. 1). One cluster, comprised of 21 samples (18 patients), contained relatively high numbers of activated dendritic cells and Th1 cells (activated immune microenvironment), whereas a second cluster, which included 20 samples (14 patients), contained increased inactive dendritic cells and basophils (inactivated immune microenvironment). Correlating with the above findings, dendritic costimulatory molecules and TH1 cell markers showed higher expression in the activated group (**Supplementary Fig. 1**).

The clinicopathologic features of the two subgroups are summarized in **Supplementary Fig. 2**. Patient demographics, tissue site (primary versus metastasis as well as liver vs non-liver metastasis), tumor grade and stage at diagnosis did not significantly differ between the groups, though right-sided tumors were more common in the suppressed immune group (p = 0.019). In addition, molecular analysis revealed more frequent KRAS mutations in the suppressed subgroup (75% versus 38%, p = 0.03) In keeping, CMS3 subtype was more common in the suppressed tumors while CMS2 subtype predominated in the activated group.

**Figure 2:**
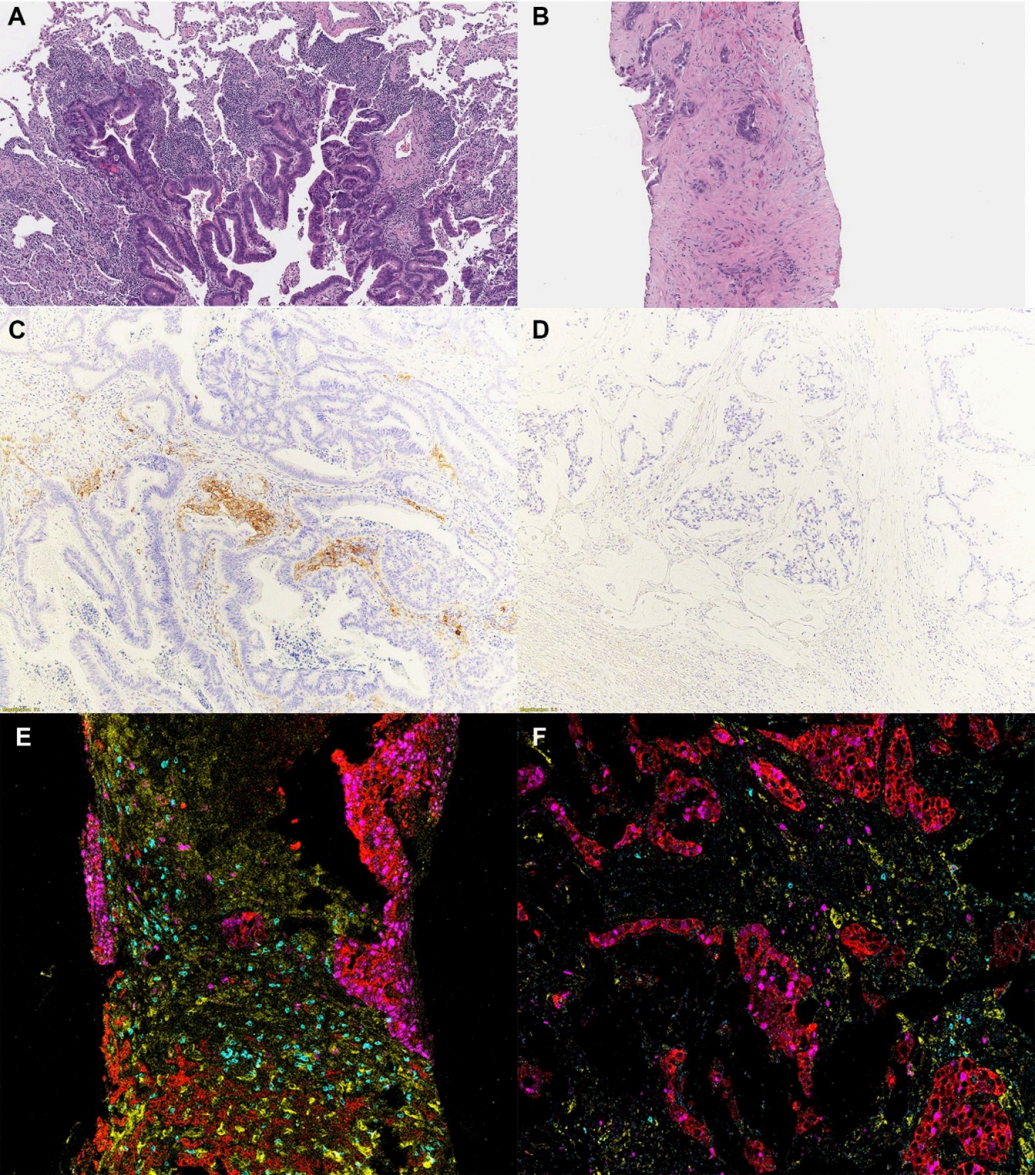
Review of the tumor morphology by H&E (a,b) and Imaging Mass Cytometry (e,f) revealed a trend towards tertiary lymphoid structures in the immune activated (cluster C1) tumors (a,c,e) whereas those with inactive immune environment (cluster C2) often harbored dense, pauci-inflammatory stroma (b,d,f). Immunohistochemical staining for CD86 demonstrates positivity in the immune cells for an immune activated tumor (c), whereas a tumor with an inactivated immune microenvironment is negative (d). Imaging Mass Cytometry revealed an increase in CD8 T cells within the tumor in cases of an activated immune microenvironment (e), whereas those with an inactivated immune microenvironment harbored increased macrophages (f). H&E = Hematoxylin and eosin. Color Key (e,g): Red-PanCK, Magenta-Ki67, Cyan-CD8 (T cells), Yellow-CD16 (macrophages).

We then sought to confirm and further characterize these two immune subtypes by detailed histopathologic review and by Imaging Mass Cytometry. Fifteen samples (8 activated and 7 suppressed) underwent detailed histopathologic review. There was no difference in tumor architecture or histologic subtype, tumor budding, or necrosis. The presence of neutrophils, eosinophils, and plasma cells did not significantly differ between the two groups. However, tertiary lymphoid structures (TLS), by microscopic identification, were present in 5 tumors analyzed, 4 of which were categorized as activated. While these were numerous in two cases (both activated), most contained occasional TLS within the tumor periphery (Fig. 2a). In addition, a dense stromal response was present in 5 (71%) of the suppressed tumors whereas this response was only seen in 1 (14%) tumor within the activated group (p = 0.029) (Fig. 2b). Immunohistochemical staining for CD80 and CD86 (activated dendritic cells markers) was performed in representative cases, which showed strong positivity at the tumor stromal interface in activated tumors but not in suppressed tumors (Fig. 2c,d), supporting the RNA-Seq findings.

**Figure 3:**
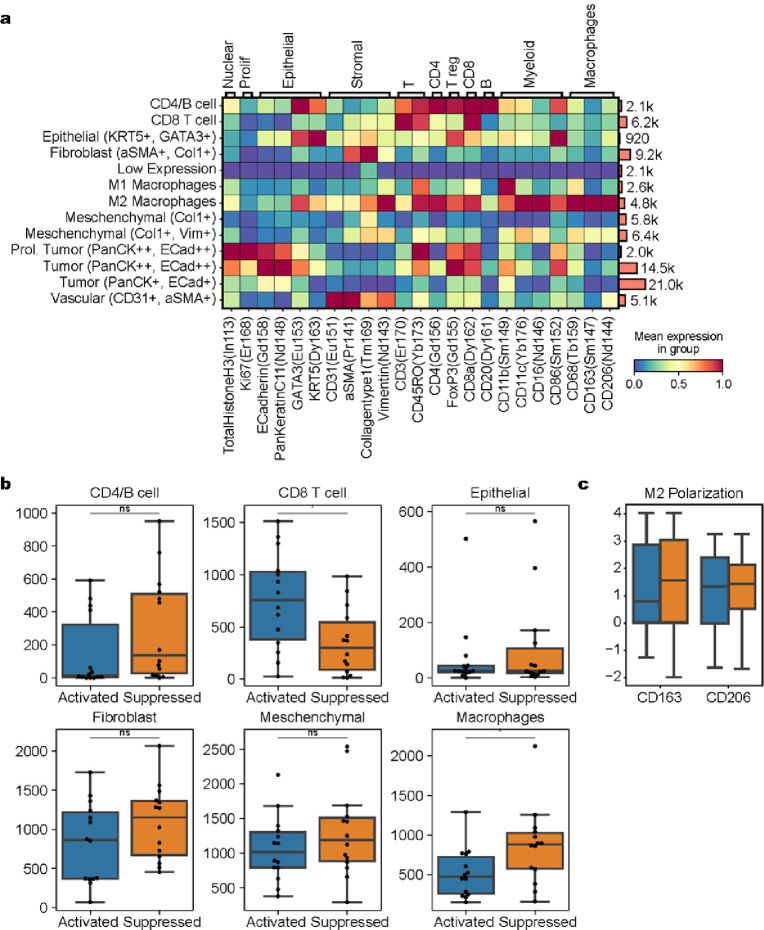
**a.** Quantitative analysis of Imaging Mass Cytometry demonstrates that CD8 T cells and macrophages were the most abundant inflammatory cells in the colorectal carcinoma cohort. No significant difference in cell type proportion was noted across primary or metastatic sites. **b.** When comparing activated versus inactivated tumors by transcriptomic analysis, we found significant differences in the cell composition of the TIME between the two groups, with CD8 T cells being more frequent in the activated subgroup whereas macrophages (predominantly M2 subtype, **c**) were more often seen in the inactivated group. TIME = Tumor immune microenvironment.

We focused the multiplexed IMC^™^ analysis on the immune composition of each tumor. To quantify the in situ expression of 26 protein markers, IMC^™^ was performed on a total of 83,898 cells from 15 tumors (11.14 mm^2^, 35 regions of interest). The antibodies included in the panel are listed in **Supplementary Fig. 1**and Fig. 3a. Qualitatively, IMC^™^ demonstrated a more robust T cell infiltrate throughout the peritumoral stroma within the activated group, with increased TLS, as was demonstrated on the hematoxylin & eosin-stained tissue slide. In suppressed tumors, the dense stromal response was highlighted, as well as an increased in macrophages (Fig. 2e,f). Quantitatively, CD8 T cells and macrophages were the most abundant inflammatory cells in the cohort (Fig. 3a, **right panel**). When comparing activated versus suppressed tumors, we found significant differences in the cell composition of the tumor microenvironment, with CD8 T cells being more frequent in the activated subgroup whereas macrophages were more often seen in the suppressed group, with increased M2/M1 ratio in the suppressed subgroup and increased expression of CD163 and CD206 (Fig. 3b,c). Dendritic cells were difficult to isolate through this methodology and the activity state could not be reliably determined, as signals for CD86 and CD80 by this methodology were weak and relatively nonspecific.

**Figure 4:**
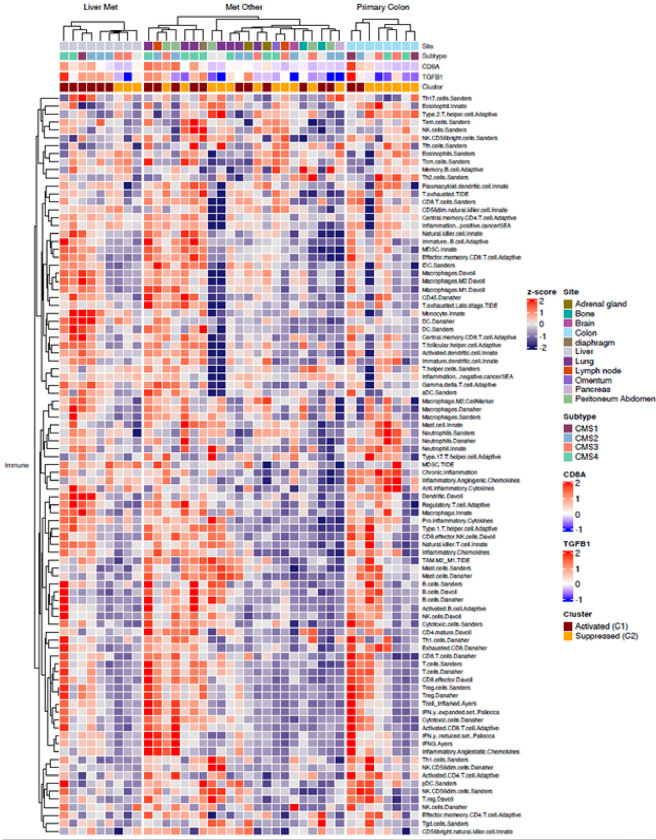
Gene expression heatmap of the tumor immune microenvironment (TIME) of primary and metastatic colorectal carcinoma (CRC) aligned by CMS molecular classification and anatomic site. The expression value is log-transformed and median centered for selected genes. Assigned activated and suppressed clusters are represented on top. Supervised consensus clustering of our CRC tumors according to a 170-immune gene signature classifies tumors into cluster C1 that contains a higher number of activated dendritic cells and Th1 cells (activated TIME), and cluster C2 that contains increased inactive dendritic cells and basophils (inactivated TIME).

We next examined the immune microenvironment as it relates to tumor location or metastases site. Although the IMC analysis identified no significant difference in cell type proportion across primary or metastatic sites (Supplemental Table 2), we did observe that the immune activated v immunosuppressed clusters readily separate in both the primary tumor and in colorectal liver metastases clusters, whereas this distinction was less apparent with extrahepatic CRC metastases (Fig. 4). In patients with CRC liver metastases, we observe an inflammatory TIME enriched in CD8 and CD4 T cells and M1 macrophages, along with pro-tumor immune infiltrate of M2 macrophages, MDSCs and Tregs. The immune suppressed CRC liver metastasis cluster is also enriched in TGFb signaling and the immune checkpoint blockade signature 2 (Fig. 4), consistent with the observation that active liver metastases induce systemic immunosuppression and relative resistance to immunotherapy. Four patients had samples from multiple sites that underwent RNA-Seq analysis. Three patients had 2 samples each, whereas metastases from 5 distinct sites as well as the primary tumor were evaluated in one patient (**Supplementary Table 3**). In two patients, the metastatic tumors clustered similarly. However, in one patient the initial primary carcinoma was found to have a suppressed immune environment while the subsequent lung metastasis demonstrated an activated immune environment. Additionally, for the patient with 6 distinct tumor samples, all but one clustered as suppressed while the adrenal metastasis was classified as activated.

### Distinct tumor immune microenvironments of colorectal carcinoma are associated with overall survival

**Figure 5:**
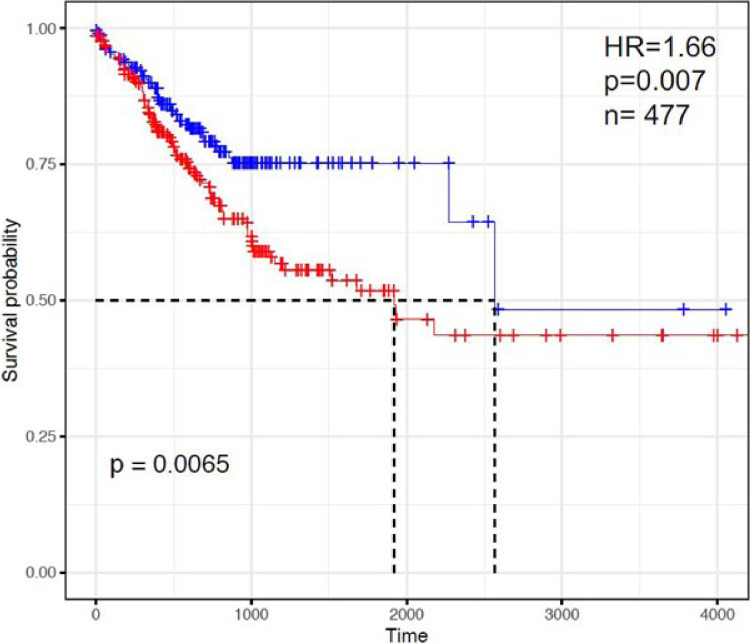
Survival probability in relation to the dendritic cell ratio. Inactive dendritic cells in CRC (red) had a lower overall survival probability compared with more activated dendritic phenotype (blue).

To examine the activated and immune suppressed TIME CRC signature in a larger cohort, a similar gene set enrichment analysis with unsupervised clustering was performed on the publicly available RNA-Seq data from the TCGA primary colon carcinoma cohort, comprising 477 patients with newly diagnosed colorectal carcinoma. In contrast to our study cohort, these patients presented mostly with earlier stage disease and had not received therapy prior to sample acquisition. However, similar to the findings in our cohort, unsupervised clustering did separate the TCGA tumors into two separate groups, which varied predominantly based on their inactivated to activated dendritic cell ratio (**Supplementary Fig. 2**). There was no significant difference in stage of disease between the two groups, though we did again identify a significant association between KRAS mutational status and increased inactivated dendritic cells (p = 0.006). Finally, we looked at the survival probability in relation to the dendritic cell ratio. Those with increased inactive dendritic cells (i.e. the immune suppressed TME) had a lower overall survival probability compared with those who had more activated dendritic cells (i.e. activated TME) (Fig. 5, HR 1.66, p = 0.007).

## Discussion

Herein, we identify two distinct clusters of advanced colorectal carcinomas with unique immune microenvironment signatures. Our findings suggest that this distinction is based predominantly on activation states of the immune cell composition, and largely independent of the CMS subtype or of driving oncogenic mutations. This study adds to the expanding literature which focuses on the importance of the tumor microenvironment in disease heterogeneity. Using currently available advanced technology, we demonstrate, similar to the findings by Galon *et al.* in this cancer subtype, that an activated immune environment (including CD8 T cells but also dendritic cells, tertiary lymphoid structures, among others) may be associated with improved patient prognosis. Further studies utilizing these techniques to better characterize the activation state of various immune cell subpopulations will help to uncover whether tumors with activated immune environments may be more sensitive to checkpoint inhibition, offering additional therapeutic implications.

The tumor microenvironment comprises a complex network of cell types such as lymphocytes, dendritic cells, myeloid cells, fibroblasts, endothelial cells and extracellular components (cytokines, chemokines, etc.), all of which heavily influence the ability of a tumor to grow and disseminate [23–25]. The characteristics of this environment are thought to have significant prognostic and even therapeutic implications in numerous cancer subtypes including ovarian, colorectal, lung and breast, and several studies have shown that the inflammatory network in particular plays a pivotal role in the evolution of cancer [26–28]. However, it is not just the density and cell type but also a cell’s specific phenotype which remains imperative in directing the immune response to be either immunostimulatory or immunotolerant, a feature that is becoming more evident as our ability to characterize these components advances. Although we demonstrate that an activated CRC TIME is associated with improved survival, it is not clear at this time if this improved survival is due to improved benefit with cytotoxic therapy, or rather a feature of a less aggressive CRC phenotype.

Cancer cells have evolved multiple mechanisms to escape immune surveillance, such as defects in antigen presentation machinery, upregulation of negative regulatory pathways, and the recruitment of immunosuppressive cell populations which ultimately result in inefficient cytotoxic response. Our ability to characterize and define the TIME has significantly evolved. Earlier studies relying on manual inspection of glass slides and single surface marker analysis by immunohistochemistry were limited in their ability to assess the dynamic nature of the TIME. This lack of specificity in defining subpopulations of cells may account for some of the inconsistencies in the literature between cell type and prognosis. Due to the complexity of the immune microenvironment that we now recognize, computational algorithms using bulk transcriptome data have become a popular approach to better understanding this interaction between tumor and host. The CMS classification scheme, for example, was one of the first subclassification studies using RNA-Seq data in colorectal carcinomas to incorporate features of the immune microenvironment in addition to molecular alterations within the tumor, in order to derive subclassifications [6]. This is pertinent in colorectal cancer, as multiple studies have demonstrated a correlation between the presence of mature T cells and dendritic cells and a more favorable prognosis, the former being the basis for the Immunoscore [9,12,13,29,30]. Similarly, the presence of B-cell and T-cell aggregates, also known as tertiary lymphoid structures (TLS) in the TIME, is considered a favorable prognostic feature as they promote T cell activation and antitumor effects[8,31]. Dendritic cells also play a prominent role in tumor response, as they vary significantly in their immunostimulatory or immunosuppressive activity at different stages of cancer progression and are incredibly dependent on local signaling [32,33]. In general, immature dendritic cells are peripherally located and non-mobile but are highly phagocytic and can be stimulated by various factors (including tumor antigens) to undergo maturation and activation, which then elicits an immune response by presenting antigens to T cells, inducing cytotoxic T lymphocyte immune response [34]. They also secrete chemokines that cause NK cells and T cell migration into the tumor and cytokines that maintain cytotoxic functions [35,36]. Not surprisingly, studies in a variety of solid tumors have found that increased dendritic cells, particularly those in the mature state with higher expression of costimulatory molecules including CD40, CD83, and CD86, are associated with improved prognosis [37–40]. On the other hand, tumor-associated dendritic cells have been found to often exhibit impaired function, including decreased uptake and presentation of antigens, reduced expression of costimulatory surface molecules, and inefficient migration, all of which may in reduced immune response and tumor evasion [41]. Studies have shown that tumor cells can release cytokines such as IL-6, IL-10 and TGF-B that cause dendritic cells to remain in an immature or immunosuppressive stage [33,42–44]. There are also metabolic inhibitors including IDO and arginase, which are produced by tumor associated macrophages (particularly the M2 phenotype) and upregulation of receptors such as CTLA4 and PD-1, all of which may be contributing to tumor immune escape [45,46].

The findings of the current study are unique in that we were able to evaluate a specific cohort of patients with advanced colorectal carcinoma, mostly Stage IV, who had already failed first line therapy. Within this cohort, we first identified two distinct groups through unsupervised clustering that differed predominantly in the activation state of the dendritic cells and other immune cells. Our findings are in keeping with other studies which have found an association between increased Th1 and activated dendritic cells and improved prognosis, whereas increased inactivated dendritic cells are generally associated with progression of disease [7,11,47]. Prior research has also characterized KRAS mutant CRC as having limited cytotoxic T cell infiltration, reduced T helper 1 responses, and reduced INF-gamma signaling, generating an overall immunosuppressive tumor microenvironment phenotype, which correlates with our finding of increased KRAS mutations in the subgroup with a suppressed TIME [48,49]. Another unique finding of the current study is that the reactivity state of the TIME did not depend on site of disease, either primary versus metastasis or location of metastasis. This is an important point, as prior studies have suggested that liver metastases are associated with immunosuppression, and patients with liver metastases have been shown to have minimal response to systemic immunotherapy [50]. However, we identified metastatic liver tumors with both activated and suppressed TIME, suggesting that a subset of patients with hepatic involvement may benefit from immunotherapy.

There are several recognized limitations of the current study. The cohort of patients with advanced colorectal carcinoma in which sufficient material available for RNA-Seq was relatively small. In addition, while review of the histomorphology and IMC^™^ analysis were performed in order to confirm our findings in situ, this comparison was somewhat limited by the fact that the FFPE tissue areas used for histopathology, IHC and IMC^™^ analysis may not exactly correspond to matched frozen tissue areas used for bulk RNA-Seq. Second, the IMC^™^ analysis was limited to discrete regions of interest that may not be representative of the entire tissue sample. Lastly, we were unable to fully characterize the immune population through IMC^™^ given the lack of additional immune markers in the available panel. Additional studies that can isolate dendritic cells and activations states are warranted.

In conclusion, this study supports the pertinent role of the immune microenvironment in tumor progression and patient prognosis, even within a group of patients with advanced stage disease who have failed multiple lines of therapy. We characterized the immune cell composition utilizing novel, more advanced technologies such as RNA-Seq and IMC^™^ in order to better understand the complexity and vital role that activity states play in determining response to tumor infiltration, thereby affecting response to therapy and overall patient outcome.

## Figures and Tables

**Table 1:** Clinicopathologic features of the study cohort.

	Study cohort samples (N = 41)	%
Site		
Primary	9	22
Metastatic	32	78
Mismatch Repair		
MSS	39	95
MSI	2	5
Histologic grade		
Low-grade	18	44
High-grade	24	59
Molecular alterations		
KRAS mutant	23	56
APC mutant	21	51
TP53 mutant	22	54
Consensus Molecular Subtype (CMS)		
CMS1	3	7
CMS2	11	27
CMS3	9	22
CMS4	10	24
	Study cohort patients (N = 31)	
Gender (M:F)	21:10	
Mean age at diagnosis (years)	55	
Primary Location		
Right colon	6	19
Left colon	10	32
Rectum	14	45
NA	1	3
Stage at Diagnosis		
Stage I	1	3
Stage II	1	3
Stage III	17	55
Stage IV	12	39

## Data Availability

The datasets used and/or analysed during the current study are available from the corresponding author on reasonable request.

## Translational Relevance

There is a growing understanding that the tumor immune microenvironment plays a significant role in colon cancer pathogenesis, response to therapy, and overall prognosis. Herein, we utilize gene set enrichment analysis from bulk RNA-seq and multiplexed Imaging Mass Cytometry data on a cohort of advanced colorectal cancer to better understand variability in the tumor microenvironment and how this may relate to molecular findings and patient prognosis. Through unsupervised clustering of the inferred tumoral immune composition, we identify two distinct groups of tumors, those with an activated tumor microenvironment with prominent activated dendritic cells and TH1 cells and those with an inactivated tumor microenvironment. These tumor phenotypes correlate with patient outcome as those harboring activated immune components have significantly higher survival probability, validated in a publicly available cohort of colorectal cancer.

## References

[1] Siegel RL, Miller KD, Fedewa SA, Ahnen DJ, Meester RGS, Barzi A, Colorectal cancer statistics, 2017. CA Cancer J Clin 2017;67:177–93. 10.3322/caac.21395. 28248415

[2] Shibutani M, Maeda K, Nagahara H, Fukuoka T, Iseki Y, Matsutani S, Tumor-infiltrating Lymphocytes Predict the Chemotherapeutic Outcomes in Patients with Stage IV Colorectal Cancer. In Vivo 2018;32:151–8. 10.21873/invivo.11218. 29275313 PMC5892643

[3] Ryuk JP, Choi G-S, Park JS, Kim HJ, Park SY, Yoon GS, Predictive factors and the prognosis of recurrence of colorectal cancer within 2 years after curative resection. Ann Surg Treat Res 2014;86:143–51. 10.4174/astr.2014.86.3.143. 24761423 PMC3994626

[4] Lieberman DA, Rex DK, Winawer SJ, Giardiello FM, Johnson DA, Levin TR. Guidelines for colonoscopy surveillance after screening and polypectomy: a consensus update by the US Multi-Society Task Force on Colorectal Cancer. Gastroenterology 2012;143:844–57. 10.1053/j.gastro.2012.06.001. 22763141

[5] Guinney J, Dienstmann R, Wang X, de Reyniès A, Schlicker A, Soneson C, The consensus molecular subtypes of colorectal cancer. Nat Med 2015;21:1350–6. 10.1038/nm.3967. 26457759 PMC4636487

[6] Cancer Genome Atlas Network. Comprehensive molecular characterization of human colon and rectal cancer. Nature 2012;487:330–7. 10.1038/nature11252. 22810696 PMC3401966

[7] Galon J, Costes A, Sanchez-Cabo F, Kirilovsky A, Mlecnik B, Lagorce-Pagès C, Type, density, and location of immune cells within human colorectal tumors predict clinical outcome. Science 2006;313:1960–4. 10.1126/science.1129139. 17008531

[8] Fridman WH, Pagès F, Sautès-Fridman C, Galon J. The immune contexture in human tumours: impact on clinical outcome. Nat Rev Cancer 2012;12:298–306. 10.1038/nrc3245. 22419253

[9] Mlecnik B, Tosolini M, Kirilovsky A, Berger A, Bindea G, Meatchi T, Histopathologic-based prognostic factors of colorectal cancers are associated with the state of the local immune reaction. J Clin Oncol 2011;29:610–8. 10.1200/JCO.2010.30.5425. 21245428

[10] Galon J, Fridman W-H, Pagès F. The adaptive immunologic microenvironment in colorectal cancer: a novel perspective. Cancer Res 2007;67:1883–6. 10.1158/0008-5472.CAN-06-4806. 17332313

[11] Angell HK, Bruni D, Barrett JC, Herbst R, Galon J. The Immunoscore: Colon Cancer and Beyond. Clin Cancer Res 2020;26:332–9. 10.1158/1078-0432.CCR-18-1851. 31413009

[12] Li T, Fan J, Wang B, Traugh N, Chen Q, Liu JS, TIMER: A Web Server for Comprehensive Analysis of Tumor-Infiltrating Immune Cells. Cancer Res 2017;77:e108–10. 10.1158/0008-5472.CAN-17-0307. 29092952 PMC6042652

[13] Dadabayev AR, Sandel MH, Menon AG, Morreau H, Melief CJM, Offringa R, Dendritic cells in colorectal cancer correlate with other tumor-infiltrating immune cells. Cancer Immunol Immunother 2004;53:978–86. 10.1007/s00262-004-0548-2. 15197496 PMC11042674

[14] Wen R, Zhou L, Peng Z, Fan H, Zhang T, Jia H, Single-cell sequencing technology in colorectal cancer: a new technology to disclose the tumor heterogeneity and target precise treatment. Front Immunol 2023;14:1175343. 10.3389/fimmu.2023.1175343. 37256123 PMC10225552

[15] Joanito I, Wirapati P, Zhao N, Nawaz Z, Yeo G, Lee F, Single-cell and bulk transcriptome sequencing identifies two epithelial tumor cell states and refines the consensus molecular classification of colorectal cancer. Nat Genet 2022;54:963–75. 10.1038/s41588-022-01100-4. 35773407 PMC9279158

[16] Beltran H, Eng K, Mosquera JM, Sigaras A, Romanel A, Rennert H, Whole-Exome Sequencing of Metastatic Cancer and Biomarkers of Treatment Response. JAMA Oncol 2015;1:466–74. 10.1001/jamaoncol.2015.1313. 26181256 PMC4505739

[17] Sailer V, Eng KW, Zhang T, Bareja R, Pisapia DJ, Sigaras A, Integrative Molecular Analysis of Patients With Advanced and Metastatic Cancer. JCO Precis Oncol 2019;3:PO.19.00047. 10.1200/PO.19.00047. PMC6778956 31592503

[18] Rennert H, Eng K, Zhang T, Tan A, Xiang J, Romanel A, Development and validation of a whole-exome sequencing test for simultaneous detection of point mutations, indels and copy-number alterations for precision cancer care. NPJ Genom Med 2016;1:16019-. 10.1038/npjgenmed.2016.19. PMC5539963 28781886

[19] Aran D, Hu Z, Butte AJ. xCell: digitally portraying the tissue cellular heterogeneity landscape. Genome Biol 2017;18:220. 10.1186/s13059-017-1349-1. 29141660 PMC5688663

[20] Rendeiro AF, Ravichandran H, Bram Y, Chandar V, Kim J, Meydan C, The spatial landscape of lung pathology during COVID-19 progression. Nature 2021;593:564–9. 10.1038/s41586-021-03475-6. 33780969 PMC8204801

[21] Alnajar H, Ravichandran H, Figueiredo Rendeiro A, Ohara K, Al Zoughbi W, Manohar J, Tumor-immune microenvironment revealed by Imaging Mass Cytometry in a metastatic sarcomatoid urothelial carcinoma with a prolonged response to pembrolizumab. Cold Spring Harb Mol Case Stud 2022;8:a006151. 10.1101/mcs.a006151. 35483877 PMC9059779

[22] Korsunsky I, Millard N, Fan J, Slowikowski K, Zhang F, Wei K, Fast, sensitive and accurate integration of single-cell data with Harmony. Nat Methods 2019;16:1289–96. 10.1038/s41592-019-0619-0. 31740819 PMC6884693

[23] Ansell SM, Vonderheide RH. Cellular composition of the tumor microenvironment. Am Soc Clin Oncol Educ Book 2013. 10.14694/EdBook_AM.2013.33.e91. 23714465

[24] Mantovani A, Allavena P, Sica A, Balkwill F. Cancer-related inflammation. Nature 2008;454:436–44. 10.1038/nature07205. 18650914

[25] Balkwill F, Charles KA, Mantovani A. Smoldering and polarized inflammation in the initiation and promotion of malignant disease. Cancer Cell 2005;7:211–7. 10.1016/j.ccr.2005.02.013. 15766659

[26] Catacchio I, Scattone A, Silvestris N, Mangia A. Immune Prophets of Lung Cancer: The Prognostic and Predictive Landscape of Cellular and Molecular Immune Markers. Transl Oncol 2018;11:825–35. 10.1016/j.tranon.2018.04.006. 29729581 PMC6050352

[27] Zhang L, Conejo-Garcia JR, Katsaros D, Gimotty PA, Massobrio M, Regnani G, Intratumoral T cells, recurrence, and survival in epithelial ovarian cancer. N Engl J Med 2003;348:203–13. 10.1056/NEJMoa020177. 12529460

[28] Denkert C, von Minckwitz G, Brase JC, Sinn BV, Gade S, Kronenwett R, Tumor-infiltrating lymphocytes and response to neoadjuvant chemotherapy with or without carboplatin in human epidermal growth factor receptor 2-positive and triple-negative primary breast cancers. J Clin Oncol 2015;33:983–91. 10.1200/JCO.2014.58.1967. 25534375

[29] Ropponen KM, Eskelinen MJ, Lipponen PK, Alhava E, Kosma VM. Prognostic value of tumour-infiltrating lymphocytes (TILs) in colorectal cancer. J Pathol 1997;182:318–24. 10.1002/(SICI)1096-9896(199707)182:3&lt;318::AID-PATH862&gt;3.0.CO;2-6. 9349235

[30] Naito Y, Saito K, Shiiba K, Ohuchi A, Saigenji K, Nagura H, CD8+ T cells infiltrated within cancer cell nests as a prognostic factor in human colorectal cancer. Cancer Res 1998;58:3491–4. 9721846

[31] Meylan M, Petitprez F, Becht E, Bougoüin A, Pupier G, Calvez A, Tertiary lymphoid structures generate and propagate anti-tumor antibody-producing plasma cells in renal cell cancer. Immunity 2022;55:527–541.e5. 10.1016/j.immuni.2022.02.001. 35231421

[32] Hargadon KM. Tumor-altered dendritic cell function: implications for anti-tumor immunity. Front Immunol 2013;4:192. 10.3389/fimmu.2013.00192. 23874338 PMC3708450

[33] Ma Y, Shurin GV, Peiyuan Z, Shurin MR. Dendritic cells in the cancer microenvironment. J Cancer 2013;4:36–44. 10.7150/jca.5046. 23386903 PMC3564245

[34] Engelhardt JJ, Boldajipour B, Beemiller P, Pandurangi P, Sorensen C, Werb Z, Marginating dendritic cells of the tumor microenvironment cross-present tumor antigens and stably engage tumor-specific T cells. Cancer Cell 2012;21:402–17. 10.1016/j.ccr.2012.01.008. 22439936 PMC3311997

[35] Roberts EW, Broz ML, Binnewies M, Headley MB, Nelson AE, Wolf DM, Critical Role for CD103(+)/CD141(+) Dendritic Cells Bearing CCR7 for Tumor Antigen Trafficking and Priming of T Cell Immunity in Melanoma. Cancer Cell 2016;30:324–36. 10.1016/j.ccell.2016.06.003. 27424807 PMC5374862

[36] Spranger S, Dai D, Horton B, Gajewski TF. Tumor-Residing Batf3 Dendritic Cells Are Required for Effector T Cell Trafficking and Adoptive T Cell Therapy. Cancer Cell 2017;31:711–723.e4. 10.1016/j.ccell.2017.04.003. 28486109 PMC5650691

[37] Lijun Z, Xin Z, Danhua S, Xiaoping L, Jianliu W, Huilan W, Tumor-infiltrating dendritic cells may be used as clinicopathologic prognostic factors in endometrial carcinoma. Int J Gynecol Cancer 2012;22:836–41. 10.1097/IGC.0b013e31825401c6. 22617481

[38] Gulubova MV, Ananiev JR, Vlaykova TI, Yovchev Y, Tsoneva V, Manolova IM. Role of dendritic cells in progression and clinical outcome of colon cancer. Int J Colorectal Dis 2012;27:159–69. 10.1007/s00384-011-1334-1. 22065108

[39] Iwamoto M, Shinohara H, Miyamoto A, Okuzawa M, Mabuchi H, Nohara T, Prognostic value of tumor-infiltrating dendritic cells expressing CD83 in human breast carcinomas. Int J Cancer 2003;104:92–7. 10.1002/ijc.10915. 12532424

[40] Schwaab T, Weiss JE, Schned AR, Barth RJ. Dendritic cell infiltration in colon cancer. J Immunother 2001;24:130–7. 11265770

[41] Harimoto H, Shimizu M, Nakagawa Y, Nakatsuka K, Wakabayashi A, Sakamoto C, Inactivation of tumor-specific CD8^+^ CTLs by tumor-infiltrating tolerogenic dendritic cells. Immunol Cell Biol 2013;91:545–55. 10.1038/icb.2013.38. 24018532 PMC3806489

[42] Brown RD, Pope B, Murray A, Esdale W, Sze DM, Gibson J, Dendritic cells from patients with myeloma are numerically normal but functionally defective as they fail to up-regulate CD80 (B7–1) expression after huCD40LT stimulation because of inhibition by transforming growth factor-beta1 and interleukin-10. Blood 2001;98:2992–8. 10.1182/blood.v98.10.2992. 11698282

[43] Kichler-Lakomy C, Budinsky AC, Wolfram R, Hellan M, Wiltschke C, Brodowicz T, Deficiences in phenotype expression and function of dentritic cells from patients with early breast cancer. Eur J Med Res 2006;11:7–12. 16504954

[44] Satthaporn S, Robins A, Vassanasiri W, El-Sheemy M, Jibril JA, Clark D, Dendritic cells are dysfunctional in patients with operable breast cancer. Cancer Immunol Immunother 2004;53:510–8. 10.1007/s00262-003-0485-5. 14740176 PMC11032822

[45] Rodriguez PC, Quiceno DG, Zabaleta J, Ortiz B, Zea AH, Piazuelo MB, Arginase I production in the tumor microenvironment by mature myeloid cells inhibits T-cell receptor expression and antigen-specific T-cell responses. Cancer Res 2004;64:5839–49. 10.1158/0008-5472.CAN-04-0465. 15313928

[46] Chong H, Vodovotz Y, Cox GW, Barcellos-Hoff MH. Immunocytochemical localization of latent transforming growth factor-beta1 activation by stimulated macrophages. J Cell Physiol 1999;178:275–83. 10.1002/(SICI)1097-4652(199903)178:3&lt;275::AID-JCP1&gt;3.0.CO;2-Q. 9989773

[47] Fu C, Jiang A. Dendritic Cells and CD8 T Cell Immunity in Tumor Microenvironment. Front Immunol 2018;9:3059. 10.3389/fimmu.2018.03059. 30619378 PMC6306491

[48] Blaj C, Schmidt EM, Lamprecht S, Hermeking H, Jung A, Kirchner T, Oncogenic Effects of High MAPK Activity in Colorectal Cancer Mark Progenitor Cells and Persist Irrespective of RAS Mutations. Cancer Res 2017;77:1763–74. 10.1158/0008-5472.CAN-16-2821. 28202525

[49] Lal N, White BS, Goussous G, Pickles O, Mason MJ, Beggs AD, KRAS Mutation and Consensus Molecular Subtypes 2 and 3 Are Independently Associated with Reduced Immune Infiltration and Reactivity in Colorectal Cancer. Clin Cancer Res 2018;24:224–33. 10.1158/1078-0432.CCR-17-1090. 29061646 PMC5777581

[50] Yu J, Green MD, Li S, Sun Y, Journey SN, Choi JE, Liver metastasis restrains immunotherapy efficacy via macrophage-mediated T cell elimination. Nat Med 2021;27:152–64. 10.1038/s41591-020-1131-x. 33398162 PMC8095049

